# Personalizing postpartum cardiovascular prevention after preeclampsia: Is routine statin therapy justified in normotensive survivors? A critical narrative review

**DOI:** 10.1016/j.ijcrp.2026.200725

**Published:** 2026-10-29

**Authors:** Sarah Naja

**Affiliations:** Preventive Medicine Division, Hamad Medical Corporation, Doha, Qatar

**Keywords:** Prevention, Risk assessment

## Abstract

**Background:**

Preeclampsia is a pregnancy-related cardiovascular risk-enhancing factor associated with increased lifetime cardiovascular disease (CVD) risk. However, long-term cardiovascular risk varies according to postpartum blood pressure, metabolic health, and development of conventional cardiovascular risk factors.

**Current clinical recommendations:**

Current guidelines recognize preeclampsia as a risk-enhancing factor to support cardiovascular risk assessment and shared decision-making regarding lipid-lowering therapy. However, preeclampsia alone is not an indication for routine statin therapy, and no postpartum low-density lipoprotein cholesterol (LDL-C) target is recommended solely on the basis of previous preeclampsia.

**Evidence gap:**

Existing evidence is derived mainly from observational studies involving heterogeneous populations. Few studies have evaluated women who remain normotensive with favorable metabolic profiles, and no completed randomized trial has demonstrated that postpartum statin therapy improves long-term cardiovascular outcomes.

**Main argument:**

Lifelong cardiovascular surveillance is warranted after preeclampsia, but routine statin therapy for all survivors is not supported. Lipid-lowering therapy should be individualized according to overall cardiovascular risk.

**Future directions:**

Future studies should identify high-risk phenotypes, validate postpartum risk prediction tools such as PREVENT, and determine whether selected women benefit from targeted statin therapy.

## 1 Introduction: preeclampsia as a cardiovascular risk enhancer

Preeclampsia affects approximately 2–8% of pregnancies worldwide and remains a major contributor to maternal morbidity and mortality. Beyond pregnancy-related complications, preeclampsia is associated with increased lifetime risk of chronic hypertension, ischemic heart disease, stroke, heart failure, and cardiovascular mortality [1]. Consequently, major cardiovascular societies, including the American Heart Association (AHA), American College of Cardiology (ACC), and European Society of Cardiology (ESC), recognize preeclampsia as a cardiovascular risk-enhancing factor during primary prevention assessment [2], [3].

However, an important clinical question remains: should all women with previous preeclampsia receive the same preventive strategy regardless of their postpartum cardiovascular profile? Preeclampsia is a heterogeneous syndrome. While some survivors develop persistent hypertension, obesity, diabetes, or metabolic syndrome, others recover normal blood pressure and remain free of conventional cardiovascular risk factors. Although postpartum hypertension is more common after preeclampsia, a substantial proportion of women do not develop persistent hypertension, highlighting heterogeneity in long-term cardiovascular risk [4]. Therefore, applying uniform pharmacological prevention strategies may not accurately reflect individual cardiovascular risk.

This review evaluates whether routine statin therapy is justified after preeclampsia, focusing specifically on normotensive survivors, and discusses the importance of individualized cardiovascular risk assessment.

## 2 Current cardiovascular prevention recommendations after preeclampsia

Current lipid guidelines identify preeclampsia as a risk-enhancing factor that may influence cardiovascular prevention decisions. However, this designation is intended to refine overall cardiovascular risk assessment rather than automatically mandate statin therapy [5], [6], [7]. Current recommendations emphasize evaluation of traditional risk factors, including LDL-cholesterol (LDL-C), blood pressure, diabetes status, kidney function, smoking status, family history, and overall cardiovascular risk profile.

Unlike secondary prevention populations or individuals with established atherosclerotic cardiovascular disease, there is no specific LDL-C target recommended solely because of a history of preeclampsia. Lipid-lowering therapy decisions should therefore be based on absolute cardiovascular risk and expected treatment benefit. A normotensive woman with normal metabolic parameters and low calculated risk may derive limited absolute benefit from statin therapy compared with a woman with persistent hypertension, elevated LDL-C, diabetes, or multiple additional risk factors [6], [7].

## 3 Limitations of current evidence and need for individualized assessment

Evidence linking preeclampsia with future cardiovascular disease is derived primarily from observational cohort studies. Although these studies consistently demonstrate increased cardiovascular risk after preeclampsia, interpretation is limited by heterogeneity in patient populations and incomplete characterization of postpartum cardiovascular recovery. Large meta-analyses have confirmed increased risks of hypertension, ischemic heart disease, stroke, and cardiovascular mortality after preeclampsia; however, these studies included women with diverse cardiovascular profiles and could not determine risk among healthy normotensive survivors [5], [8].

Many studies evaluating endothelial function included women with persistent hypertension, increased body mass index, insulin resistance, or metabolic abnormalities, which are independent contributors to vascular dysfunction and cardiovascular disease [9], [10], [11]. Therefore, it remains difficult to distinguish the direct long-term effects of preeclampsia from the consequences of subsequent cardiovascular risk factor development.

Longitudinal studies have also provided limited information regarding vascular recovery. Östlund et al. evaluated endothelial function after preeclampsia at approximately one year postpartum and again 11 years later, demonstrating improvement in endothelial function despite persistence of some cardiovascular risk factors. However, the lack of intermediate assessments limits understanding of the timing and extent of vascular recovery [12].

Importantly, few studies have specifically examined women who recover a normotensive and metabolically healthy phenotype after pregnancy. This evidence gap limits the ability to determine whether these women have sufficient absolute cardiovascular risk to justify routine preventive medication. Table 1 shows the available evidence.

**Table 1:** Summary of major studies evaluating cardiovascular risk, vascular function, and statin-related evidence after preeclampsia.

Study	Country	Study Design	Sample Size	Mean Age at Follow-up	Follow-up Duration	Patient Characteristics at Follow-up	Endothelial Assessment	Main Findings	Major Limitations
Agatisa et al., 2004 [9]	USA	Case–control	34	Mid-30s	∼10 years	Previous preeclampsia group had higher blood pressure, higher BMI, greater insulin resistance; most were not selected for persistent normotension	Flow-mediated dilation (FMD)	Persistent endothelial dysfunction compared with controls	Small sample; observational design; residual confounding by cardiovascular risk factors
Blaauw et al., 2007 [10]	Netherlands	Prospective cohort	143	∼40 years	11 years	Approximately half developed chronic or masked hypertension; higher prevalence of microalbuminuria and metabolic abnormalities	Endothelial function and renal markers	Increased cardiovascular risk factors persisted after preeclampsia	High prevalence of hypertension limits extrapolation to normotensive women
Östlund et al., 2013 [12]	Sweden	Prospective longitudinal cohort	31	Late 30s	1 year and 11 years	Elevated blood pressure and metabolic risk factors remained more common than controls; diabetes uncommon	Flow-mediated dilation	Endothelial function improved over time despite persistent cardiovascular risk factors	No intermediate vascular assessments between years 1 and 11; small cohort
EPOCH Study [11]	International cohort study	Cross-sectional	>200	∼37 years	Mean 5–6 years	Mixed population including normotensive and hypertensive women with varying BMI and metabolic risk	Vascular function testing	Cardiovascular phenotype after hypertensive pregnancy is heterogeneous	Cross-sectional design prevents assessment of temporal recovery
Bellamy et al., 2007 [5]	International Meta-analysis	Systematic review	>3 million women	Not applicable	Variable	Included women with diverse cardiovascular profiles	Not applicable	Previous preeclampsia associated with increased future cardiovascular disease	Observational evidence; unable to distinguish women who recovered normal cardiovascular health
Wu et al., 2017 [8]	International Meta-analysis	Systematic review	>6 million women	Not applicable	Variable	Mixed-risk populations; chronic hypertension common	Not applicable	Increased long-term risk of ischemic heart disease, stroke and heart failure	Significant heterogeneity among included cohorts
Costantine et al. (Pravastatin Trial) [13]	USA	Randomized controlled trial	High-risk pregnant women	Pregnancy	During pregnancy	Women at high risk for recurrent preeclampsia	Clinical outcomes	No convincing reduction in preeclampsia or maternal cardiovascular outcomes	Evaluated antenatal rather than postpartum statin therapy
SPARK Trial (ongoing) [14]	Canada	Phase IV randomized placebo-controlled trial	Planned ∼76	Adult postpartum women	12 months	Women with previous hypertensive disorders of pregnancy after breastfeeding; includes varying cardiovascular risk profiles	Biomarkers and vascular measures	Designed to evaluate atorvastatin for postpartum primary prevention	Designed to evaluate the effect of postpartum atorvastatin on cardiovascular risk markers; clinical outcomes are awaited.

## 4 Evidence for postpartum statin therapy after preeclampsia

Despite increasing recognition of adverse pregnancy outcomes as cardiovascular risk enhancers, no completed randomized controlled trial has demonstrated that postpartum statin therapy reduces cardiovascular events or improves long-term vascular outcomes specifically among women with previous preeclampsia.

Previous statin trials in pregnancy have primarily focused on prevention of recurrent preeclampsia rather than postpartum cardiovascular prevention. The pravastatin trial by Costantine et al. evaluated high-risk pregnant women and was not designed to assess long-term cardiovascular outcomes after delivery. Therefore, these findings cannot be directly applied to postpartum statin therapy [13].

The ongoing SPARK trial is evaluating atorvastatin therapy after hypertensive disorders of pregnancy and may provide important evidence regarding vascular biomarkers and cardiovascular risk modification. Until these data become available, routine statin therapy after preeclampsia remains based on extrapolation from general primary prevention populations rather than direct evidence [14].

## 5 Risk-based statin decision-making and Future Directions

Current cardiovascular prevention approaches support individualized decision-making rather than diagnosis-based treatment. The American Heart Association PREVENT equations estimate total cardiovascular disease risk using contemporary clinical variables; however, they have not yet been specifically validated in women with previous hypertensive disorders of pregnancy and do not include pregnancy-specific factors such as disease severity, timing of onset, or endothelial biomarkers [15].

Future research should focus on identifying high-risk phenotypes among preeclampsia survivors, including women with early-onset or severe disease, recurrent preeclampsia, persistent hypertension, metabolic abnormalities, subclinical atherosclerosis, or abnormal vascular biomarkers. Prospective studies and randomized trials are required to determine whether selected subgroups benefit from targeted pharmacological prevention.

## 6 Conclusion

Preeclampsia is an important cardiovascular risk-enhancing factor; however, its presence alone does not currently justify routine statin therapy or universal LDL-C targets in postpartum women. Most normotensive survivors with favorable metabolic profiles should receive lifestyle counseling, cardiovascular risk monitoring, and long-term surveillance rather than automatic pharmacological treatment. Conversely, women with persistent cardiovascular risk factors may benefit from earlier lipid-lowering therapy through shared decision-making.

Personalized cardiovascular prevention strategies, supported by future prospective research and randomized trials, are needed to identify which women after preeclampsia are most likely to benefit from preventive pharmacotherapy.

## Declaration of generative AI and AI-assisted technologies in the writing process

ChatGPT (OpenAI) was used for language editing and improving manuscript clarity. All AI-assisted content was reviewed and approved by the authors, who take full responsibility for the final manuscript.

## Funding acknowledgement

Publication Funded by the 10.13039/100031665Medical Research Centre (MRC) – Hamad Medical Corporation (HMC).

## Declaration of competing interest

The authors declare that they have no known competing financial interests or personal relationships that could have appeared to influence the work reported in this manuscript.

No financial support, grants, or sponsorship from commercial organizations were received for the preparation of this manuscript.

The authors confirm that there are no conflicts of interest related to the content, interpretation, or publication of this review.

## List of abbreviations:

BMI: body mass index
FMD: flow-mediated dilation
